# Soil Microbial Carbon Use Efficiency in Natural Terrestrial Ecosystems

**DOI:** 10.3390/biology14040348

**Published:** 2025-03-27

**Authors:** Weirui Yu, Lianxi Sheng, Xue Wang, Xinyu Tang, Jihong Yuan, Wenbo Luo

**Affiliations:** 1 Key Laboratory of Wetland Ecology and Vegetation Restoration, Ministry of Ecology and Environment, School of Environment, Northeast Normal University, Changchun 130117, China; yuwr@nenu.edu.cn (W.Y.); wangx881@nenu.edu.cn (X.W.); tangxinyu929@nenu.edu.cn (X.T.); 2 Key Laboratory of Vegetation Ecology, Ministry of Education, Northeast Normal University, Changchun 130024, China; 3 National Ecosystem Research Station of Jiangxi Wugong Mountain Meadow, Wetland Ecological Resources Research Center, Jiangxi Academy of Forestry, Nanchang 330032, China; yuanjh0107@jxlky.cn

**Keywords:** terrestrial ecosystems, soil microbes, microbial carbon use efficiency, environmental factors, anaerobic metabolic

## Abstract

Soil microbial carbon use efficiency (CUE) is the ratio of carbon allocated to microbial growth to that taken up by microorganisms. Soil microbial CUE affects terrestrial ecosystem processes such as greenhouse gas emissions, carbon turnover, and sequestration, which is an important indicator of changes in the terrestrial carbon cycle. Firstly, we summarized the three methods of soil microbial CUE, stoichiometric modeling, ^13^C glucose tracing, and ^18^O water tracing, and compared the advantages and limitations of the three methods. Then, we analyzed the single or combined effects of different environmental factors on soil microbial CUE in grassland ecosystems, forest ecosystems, and wetland ecosystems. Finally, we suggested that future research should focus on the following aspects: the influence of management patterns on CUE (such as grazing and the prohibition of grazing in grassland ecosystems, forest gap, and thinning in forest ecosystems); effects of the strategies of microorganisms for adapting to environmental changes on CUE; effects of anaerobic metabolic pathways, especially in wetland ecosystems; and effects of microbial taxonomic level. This study contributes to the investigation of the microbial mechanisms of carbon cycling in terrestrial ecosystems to mitigate the impacts of climate change.

## Simple Summary

Soil microbial carbon use efficiency (CUE) is an important indicator of carbon cycle changes in terrestrial ecosystems. Exploring the mechanisms by which environmental factors affect CUE in terrestrial ecosystems is important for a better understanding of the process of global carbon cycling. In this study, we summarized the advantages and limitations of the main methods for measuring CUE. After that, we analyzed the single or combined effects of different environmental factors on CUE in grassland ecosystems, forest ecosystems, and wetland ecosystems, respectively. Finally, we suggested that future research should focus on the following aspects: the influence of management patterns on CUE, effects of the strategies of microorganisms for adapting to environmental change on CUE, effects of anaerobic metabolic pathways, especially in wetland ecosystems, and effects of microbial taxonomic level. This study provides a scientific basis for the study of terrestrial soil carbon cycling under the background of global change.

## 1. Introduction

Global climate change affects the absorption and release of greenhouse gases (such as methane, carbon dioxide, etc.) in terrestrial ecosystems, thus affecting the land–atmosphere carbon exchange. The soil ecosystem, one of the most important carbon sinks in the world, stores a large amount of carbon.

**Figure 1:**
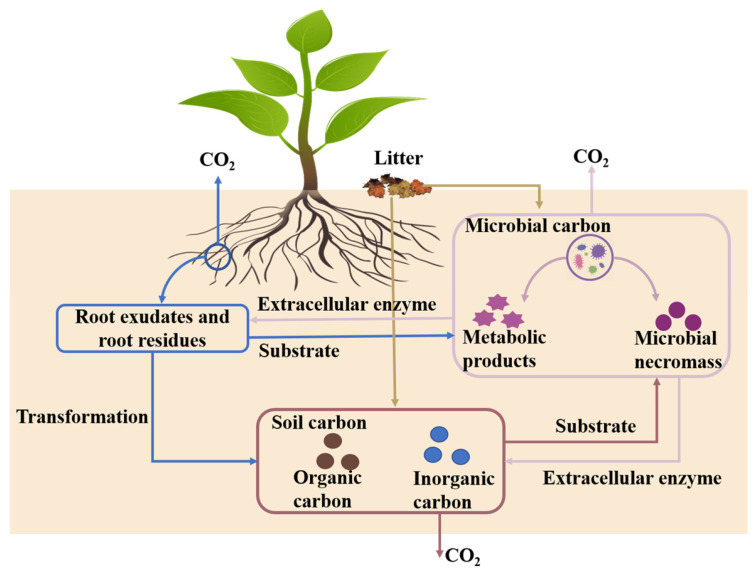
Soil material transformation processes. The blue box, brown box, and purple box indicate the vegetation, soil, and microbial carbon pool, respectively. The blue line indicates the vegetation carbon pool input into the other carbon pools, the yellow line indicates the litter input into the other carbon pools, the purple line indicates the microbial carbon pool input into other carbon pools, and the brown line indicates the soil carbon pool input into the other carbon pools.

Microorganisms participate in almost all material transformation processes in soil ecosystems [1,2,3], including material and energy transformations among plants, microorganisms, and soil (Figure 1). Part of the carbon obtained by soil microorganisms is used for their growth and remains in the ecosystems for a long time, while the other part of the carbon is emitted into the air through respiration, resulting in increased atmospheric carbon dioxide concentrations [4]. Consequently, the metabolic activity of microorganisms could affect the global carbon cycle. Soil microbial carbon use efficiency (CUE) is the ratio of carbon allocated to microbial growth to that taken up by microorganisms [5], which might be one of the important indicators of the trade-off between the growth and respiration of microorganisms [6]. Additionally, a higher CUE can promote carbon accumulation [1,7], and a lower CUE means less carbon storage in soil. Therefore, soil microbial CUE is important for better understanding the effects of microorganisms on the soil carbon cycle in terrestrial ecosystems [5,7,8], and might be essential for accurately assessing the potential of soil carbon storage [9].

Soil microbial CUE is often considered as a constant in the searches of soil carbon turnover [10,11,12]. However, soil microorganisms are sensitive to environmental changes. Abiotic factors, such as soil nutrient availability, moisture, and temperature might have direct influences on soil microbial CUE [6,13,14]. For example, soil microbial CUE tends to rise in response to higher soil nutrient availability, but it declines when soil moisture and temperature increase [15,16]. Additionally, biotic factors, such as vegetation and microbial community structure, also have been widely recognized as direct factors affecting soil microbial CUE [17,18,19,20]. The soil microbial CUE increases with the complexity of the vegetation community [21,22]. Fungal-dominated microbial communities might have higher CUE than bacterial-dominated microbial communities [23]. More importantly, abiotic factors could also indirectly affect the CUE by influencing the biotic factors [13]. For instance, elevated temperatures might reduce CUE by inhibiting microbial activities [24]. Long-term acid rain treatment significantly inhibited soil microbial activity, especially the abundance of bacteria and fungi, and led to a decrease in carbon acquisition enzymes [25], in turn affecting soil microbial CUE. However, how the environmental factors directly or indirectly affect CUE is still uncertain [5,7,13,26].

Previous related studies usually analyzed the effects of environmental factors (e.g., temperature, nutrient content, substrate quality, etc.) on soil microbial CUE [7,13,27,28,29,30]. To our knowledge, less literature has comprehensively compared the effects of environmental factors on soil microbial CUE in different ecosystems, especially in wetland ecosystems. Therefore, we conducted an extensive literature search using (a) “CUE” or “carbon use efficiency” or “microbial metabolism” and (b) “forests or “forest ecosystems” and (c) “wetlands” or “peatlands” or “wetland ecosystems” and (d) “grasslands” or “grassland ecosystems” and (e) “environmental factors” or “biotic factors” or “abiotic factors” and (f) “soil carbon cycle” or “soil carbon sequestration” as the search term, by searching the Web of Science, China National Knowledge Infrastructure, Scopus, ScienceDirect, Wiley Online Library, and Springer Links through 20 February 2025. In this paper, we summarized the measurement methods for soil microbal CUE (Table 1). Then we analyzed the biotic and abiotic factors affecting CUE in different ecosystems. Our work integrates extensive methods and findings, offering an overall perspective of the current state of studies. Finally, we provide an outlook for future research based on the results of our analysis. Our study not only contributes to the theoretical understanding of soil microbial ecology but also provides practical insights for future research directions.

## 2. Variations in Various Methods

### 2.1. Stoichiometric Modeling

Many studies have used Ecoenzymatic Stoichiometry Theory to quantify microbial metabolic activities at different scales [37,38]. Soil microbial CUE could be calculated by quantifying the extracellular enzyme activities [31,39,40], including β-1,4-glucosidase (BG), leucine aminopeptidase (LAP), β-1,4-N-acetylaminoglucosidase (NAG), and acid phosphatase (AP), which mediate the acquisition of carbon (BG), N (LAP and NAG), and P (AP) from the soil. The calculation formulae are as follows:

(1) $$\mathrm{CUE}={\mathrm{CUE}}_{\max}\times {\left(\left(S_{C:N}\times S_{C:P}\right)/\left[\left(K_{C:N}+S_{C:N}\right)\times \left(K_{C:P}+S_{C:P}\right)\right]\right)}^{0.5}$$

(2) $$S_{C:N}=B_{C:N}/L_{C:N}\times 1/{\mathrm{EEA}}_{C:N}$$

(3) $$S_{C:P}=B_{C:P}/L_{C:P}\times 1/{\mathrm{EEA}}_{C:P}$$

where K_C:N_ and K_C:P_ are the half-saturation constants for CUE based on the stoichiometry of the substrate C:N and C:P effectiveness. We assumed a K_C:N_ and K_C:P_ of 0.5 and CUE_max_ of 0.6 in this study model [41]. B_C:X_ is the elemental C:N or C:P ratio of the microbial biomass, L_C:X_ is the elemental composition of the effective substrate, and EEA_C:X_ is the ratio of enzymatic activity to obtain carbon and nutrients (X) from the environment. L_C:N_ and L_C:P_ were estimated based on the C:N and C:P. EEA_C:N_ was calculated as follows: BG/(NAG + LAP); EEA_C:P_ was calculated as follows: BG/AP.

Currently, this method is more popular for calculating soil microbial CUE, mainly because all of the required indicators, such as extracellular enzyme activities, soil microbial biomass, and soil nutrient contents could easily be obtained by common soil determination methods. However, the method also has some limitations. For example, the maximum value of soil microbial CUE determined by this method was 0.6 due to thermodynamic constraints [31], resulting in a relatively narrow measurement range when compared with other methods [31]. However, most of the soil microbial CUE values are less than 0.6 in the present studies [2,41,42], indicating that this method is suitable for calculating soil microbial CUE in the present study.

### 2.2. ^13^C Glucose Tracing

Glucose is a source of energy and a metabolic intermediate for living cells, i.e., the main energy donor for organisms, which is crucial for living organisms. We can use the ^13^C isotope tracing to track the uptake and conversion of substrates, and it incorporates labeled glucose into the microbial biomass. It is inferred by calculating the differences between the ^13^C-labeled and unlabeled molecules [28]. The microbial growth (^13^MBC) was calculated as the product of total microbial biomass (F DOC − NF DOC) and the percent of total microbial biomass labeled (% ^13^MBC):

(4) $$at\%\;MBC=\frac{\left[\left(at\%\;F\;DOC\times F\;DOC\right)-\left(at\%\;NF\;DOC\times NF\;DOC\right)\right]}{\left(F\;DOC-NF\;DOC\right)}$$

(5) %MBC13=at% MBCt−at% MBCcat% sol−at% MBCc×100

(6) MBC13=FDOC−NFDOC×%MBC13÷100

(7) CUE=MBC13MBC13+R13

where at% F DOC, F DOC, at% NF DOC, and NF DOC represent the atomic % and total carbon concentration (μg C g^−1^ soil) of fumigated (F) and non-fumigated (NF) K_2_SO_4_ extracts, respectively. At% MBCt and at% MBCc are the atom % of the sample treatments and the natural abundance control, and at% sol is the carbon abundance of added markers. ^13^R is the cumulative respiration derived from added glucose (μg ^13^CO_2_-C g^−1^ soil) using a CO_2_ flux curve assembled from all respiration rates collected from nine samples over 72 h.

The tracer is easily absorbed by microorganisms, indicating that soil microbial CUE could be quickly obtained. However, this method also has some limitations. Firstly, it does not distinguish between the concepts of microbial CUE and substrate use efficiency, as it assumes that soil organic matter metabolism is equivalent to glucose metabolism [28]. During a short period, the glucose utilized by microorganisms is not exactly equal to the substrate consumed, whereas more glucose is used for community uptake, which might lead to a higher soil microbial CUE [32]. Secondly, this method may lead to some errors in the results of soil microbial CUE because rapid glucose metabolism could cause an initially higher soil microbial CUE. The ^13^C tracer in this method accumulates in an unextractable form within 6 h and is converted into mineral-stabilized microbial products. The underestimation of microbial uptake (MBC + R) consequently leads to an overestimation of CUE [32,33]. Finally, microorganisms could use different kinds of substrates to grow, indicating that glucose is not the only food source for microorganisms. More importantly, the same microorganism will have different CUE using different substrates [43,44,45], so the method of calculating from glucose may be not accurate.

### 2.3. ^18^O Water Tracing

This method could separate the DNA of active microorganisms from dead or dormant microorganisms. The total microbial growth was determined by incorporating ^18^O-labeled water into microbial DNA and extracting and quantifying the abundance of the labeled versus unlabeled DNA molecules after a short period of incubation to calculate the CUE [8,46]:

(8) $${\mathrm{DNA}}_{\mathrm{produced}}=O_{\mathrm{total}}\times \frac{{\mathrm{at}\%}_{\mathrm{excess}}}{100}\times \frac{100}{{\mathrm{at}\%}_{\mathrm{final}}}\times \frac{100}{31.21}$$

where O_total_ is the total O content (μg) of the dried DNA extracts, at%_excess_ is the difference between the O content of the labeled samples and that of the unlabeled samples, and 31.21 is the mean percentage of O in the DNA (C_39_H_44_O_24_N_15_P_4_). at%_final_ is the ^18^O at% of the soil moisture at the start of the incubation. It was also assumed that the O in the new DNA was derived from water only. Due to the short incubation time, the mortality of the newly produced ^18^O-labeled microbial cells was negligible in the experiment. A conversion factor (f_DNA_) was calculated for each sample to represent the ratio of soil MBC to soil DNA content (μg^−1^ soil). The microbial growth rate (growth, μg C g^−1^ soil h^−1^) was calculated by multiplying DNA yield and f_DNA_:

(9) $$\mathrm{Growt}h=\frac{f_{\mathrm{DNA}}\times {\mathrm{DNA}}_{\mathrm{produced}}\times 1000}{\mathrm{DW}\times t}$$

where DW (g) is the soil dry weight and t is the incubation time (h). In addition, the microbial respiration rate (respiration, μg C g^−1^ soil h^−1^) was calculated by the following equation:

(10) $$\mathrm{Respiration}=\frac{R_{s}}{\mathrm{DW}\times t}\times \frac{p\times n}{R\times T}\times V\times 1000$$

where p is the atmospheric pressure (kPa), n is the molecular mass of element C (12.01 g mol^−1^), R is the ideal gas constant (8.314 J mol^−1^K^−1^), T is the absolute temperature of the gas (295.15 K), and V is the headspace volume of the vials (L). R_s_ (ppm) is the concentration of CO_2_ produced during the 24 h incubation.

(11) $$\mathrm{CUE}=\frac{C_{\mathrm{growt}h}}{C_{\mathrm{growt}h}+\mathrm{Respiration}}$$

Microbial uptake water and microbial growth occur simultaneously. The method of ^18^O water tracing provides a direct measurement of microbial growth. The soil microbial CUE value is relatively stable over time [32]. However, the method assumes water is the only source of O_2_ during the period of microbial growth, which might lead to inaccurate results. For example, Hungate et al. showed that 33% of oxygen in microbial DNA derived from water [32,35], which suggested that microorganisms could actually obtain oxygen through other pathways as well. Thus, the method might cause the soil microbial CUE to be underestimated [36].

Overall, soil microbial CUE primarily depends on whether microorganisms are in growth or decay. Soil microbial CUE increases during microbial growth and decreases during microbial decay. As a result, soil microbial CUE is to some extent transient. Based on the characteristics of soil microbial CUE, none of the current methods for determining it seem to be accurate. Therefore, more advanced methods should be developed.

## 3. Terrestrial Ecosystems

### 3.1. Grassland Ecosystems

As one of the major soil carbon pools in terrestrial ecosystems [47,48,49], grassland ecosystems contain about 30% of the global soil carbon stock [50]. Exploring soil microbial CUE and its influencing mechanisms is important to predict the responses of the carbon cycle in grassland ecosystems in global climate change conditions [51,52,53,54,55,56]. Most of the related studies have focused on the effects of nutrient addition, land management patterns, and global climate change on soil microbial CUE [51,53,57,58].

Since the Industrial Revolution, the concentrations of N and P have increased as fossil fuel burning and fertilizer application have increased. It is meaningful to investigate the effects of N and P addition on soil microbial CUE in grassland ecosystems [8,57,59,60]. N addition could affect microbial metabolism, which might have direct effects on soil microbial CUE [61]. For example, Spohn et al. found that N addition may inhibit oxidase activity in the soil and led to decreased microbial carbon uptake, whereas the total amount of carbon used for the growth of microbes remained constant during the metabolic process, with soil microbial CUE increased [8]. N addition can also affect soil microbial CUE by influencing soil microbial community structure [62]. For example, Riggs and Hobbie found that N addition might result in the higher proportion of bacteria than fungi in the soil microbial community [61]. Importantly, bacteria usually have lower soil microbial CUE than fungi (CUE_bacteria_ < CUE_fungi_) [61], so N addition can also decrease soil microbial CUE. P is also an important element for synthesizing cells, metabolism, and energy transfer, affecting microbial activity and thus, soil microbial CUE. P addition could alleviate nutrient limitations of microorganisms and thus increase soil microbial CUE [7]. However, Widdig et al. found that the addition of P has insignificant effects on soil microbial CUE, indicating that it is still unclear how P affects soil microbial CUE [57]. More importantly, the combined effects of N and P addition together on soil microbial CUE might be even more complicated than the results of single nutrient element addition, which may be positive or insignificant. For example, Poeplau et al. confirmed that nutrient addition might increase soil microbial CUE by increasing the availability of other nutrient elements in the soil [14], whereas Widdig et al. found that nutrient addition has insignificant effects on CUE because it did not cause microbial overflow respiration [57]. Therefore, we still do not know the combined effects of multiple nutrient elements addition on soil microbial CUE.

Grazing is the main form of grassland utilization. Grazing could reduce both above- and below-ground plant biomass [63,64] and decrease plant carbon inputs, microbial biomass, and activity [65]. Meanwhile, mechanical stress due to trampling by livestock could also alter soil bulk [65], which might increase the proportion of anaerobic bacteria in the soil [66,67]. All of the above affect soil microbial CUE. However, overgrazing might have severe effects on grassland ecosystems, such as the reduction of soil fertility or degradation of vegetation. Therefore, the management strategy of the prohibition of grazing is proposed to maintain grassland ecosystem functions [68]. The prohibition of grazing might have significant effects on soil microbial CUE through increasing the proportion of smaller macroaggregate, which play fundamental roles in soil nutrient maintenance, availability, and transformation [69]. A key character of smaller macroaggregates (2–0.25 mm) is resource limitation, which could limit microbial growth, and then decrease soil microbial CUE [53].

Additionally, global climate change is also a crucial factor affecting soil microbial CUE in grassland ecosystems, such as warming, which might affect grassland ecosystems through two aspects, temperature increase and drought. Generally, temperature increase has negative effects on soil microbial CUE. Microorganisms might allocate more energy to maintaining their growth or gaining more energy for survival in such conditions [28,70]. Meanwhile, microbes might allocate more carbon for respiration rather than microbial growth, which could lead to a lower soil microbial CUE [7,71], whereas for drought, it could the reduce growth of microorganisms, induce microbial dormancy (microorganisms reduce their metabolism and exist as spores, cysts, or persisters) by reducing water content, influencing infiltration potential and substrate diffusivity in soil ecosystems, which might in turn affect soil microbial CUE [43,72,73]. Drought could also affect soil microbial CUE by reducing microbial respiration [73]. For example, Fuchslueger et al. found that drought reduces microbial respiration and uptake of substrates simultaneously, which have insignificant effects on soil microbial CUE [51]. More importantly, temperature increase and drought tend to occur simultaneously, which might have combined impacts on CUE [51,58]. Both temperature increase and drought could also accelerate microbial N limitation, inhibit soil enzyme activity, and reduce soil microbial CUE [51,58]. However, the effects of temperature increase and drought on soil microbial CUE have not shown the same tendency. For example, Fuchslueger et al. confirmed that when temperature increases and drought occur simultaneously, the variations in soil microbial CUE are more dependent on the negative effects induced by temperature increase [51].

### 3.2. Forest Ecosystems

The CUE in forest ecosystems reflects the carbon assimilation capacity and carbon sink potential [74], which is an important ecological factor when exploring the response and adaptation mechanisms of forest ecosystems facing global climate change [55]. Soil microbial CUE in forest ecosystems is influenced by environmental factors such as elevation [75], temperature [76], precipitation [76], etc.

The elevation variations might have negative effects on soil microbial CUE [77]. For example, Feng et al. confirmed that the soil microbial CUE could decrease with increasing elevation in the Tibetan Plateau area [75]. Generally, the following aspects may be related to mechanisms of how the elevation variations affect soil microbial CUE. The first is soil microbial community structure [77]. Microbial communities in higher elevation areas are adapted to low temperatures and low nutrient conditions [78,79]. Feng et al. explored the role of microbial taxa with different trait strategies in regulating soil microbial CUE under different elevation gradients on the Tibetan Plateau and categorized the microbes into four microbial taxa [75]. The results suggest that the shift in the abundance of different microbial taxa in the bacterial community might be one of the key factors on the negative effects of increasing elevation on soil microbial CUE; the second is the resource allocation pattern. Microorganisms might have stronger resource constraints with increasing elevation, indicating that microorganisms could allocate more energy for limited resource acquisition (N and P) and less energy for growth, causing a lower soil microbial CUE [75]. The last is temperature, which always decreases as elevation increases. A low temperature in high elevation areas could inhibit microbial growth and metabolic activity, resulting in a decrease in the soil microbial CUE in the high elevation regions [75].

How warming affects soil microbial CUE in forest ecosystems seems to be more complicated. Warming increases forest ecosystems’ temperature, indicating the soil microbial CUE might decrease with temperature increases. For example, Steinweg et al. found that soil microbial CUE decreased by approximately 0.009 per 1 °C increase in temperature [24]. There might be two reasons for the negative relationships between temperature and soil microbial CUE. One is the energy allocation strategy of microbes. In higher temperature conditions, microbes allocated more energy to mitigate the stress of higher temperatures on the microbes themselves, thus limiting microbial growth. Another explanation is that the rate of microbial respiration increases more than the rate of growth with increasing temperature.

Precipitation can be affected by the vegetation canopy interception in forest ecosystems, which might alter the soil water availability, and then indirectly affect soil microbial CUE [55]. How precipitation affects CUE might be explained by the following aspects. Firstly, precipitation alters the infiltration environment required for the growth of soil microorganisms [80]. Secondly, precipitation also affects microbial uptake and the utilization of nutrients by affecting nutrient solubility, diffusion, and transport in the soil, which in turn affect microbial uptake and the utilization of nutrients and then affects soil microbial CUE. Finally, precipitation may inhibit microbial activity by reducing oxygen content in the soil. For instance, Wang et al. found that microbial respiration increased with increasing precipitation, which means microorganisms allocated less carbon for growth, and then CUE declined [76].

### 3.3. Wetland Ecosystems

There is about 350–535 Gt carbon in wetland ecosystems [81,82]. Unlike other ecosystems, soil carbon in wetlands primarily exists in the form of peat. Peat consists mainly of partially decomposed plant residues that accumulate over periods of time under waterlogging and anaerobic conditions [83]. This is the difference and uniqueness of the wetland ecosystems. The changes in carbon stocks in wetlands might have significant effects on the global carbon cycle [84]. Therefore, soil microbial CUE studies in wetlands are also very important, and could assist in accurately assessing the effects of wetland ecosystems on the global carbon cycle [9].

Water is a key factor affecting the microbial growth in wetland ecosystems. Oxygen diffuses very slowly through water [85], indicating that microorganisms are often under anaerobic conditions in wetland ecosystems. These conditions could inhibit microbial metabolic activity, then decrease soil microbial CUE [6,7,86]. For example, Bastviken et al. found that the soil microbial CUE was lower under anoxic conditions (CUE = 0.19) than under aerobic conditions (CUE = 0.48) [87]. There might be two explanations for this phenomenon. One is the inhibiting effect of anoxic conditions on microbial activity. For example, Picek et al. confirmed that high water content could reduce the soil microbial CUE by decreasing the oxygen concentration and then inhibiting microbial activity [88]. Another explanation is the resource allocation capacity of microorganisms. Due to the cellular permeability mechanism, soil microorganisms have to expend higher metabolic costs for survival [89], indicating that microbes might reduce the amount of energy allocated to growth, and allocate more energy to respiration [7,90], causing the reduction of soil microbial CUE.

The majority of research on soil microbial CUE in wetland ecosystems has focused on evaluating soil microbial CUE among different wetland types. For example, a meta-analysis conducted by Hill et al. showed that the soil microbial CUE varied from 0.056 to 0.302 among freshwater and estuarine wetlands [42]. In addition, Zhai et al. found that the negative relationships between salinity and soil microbial CUE might mainly depend on the trade-off mechanism regarding the transfer of energy from soil microbial CUE to extracellular enzyme production, induced by the combined inhibiting effects of salinization and P limitation on microorganism activities in tidal wetlands [2]. Taken together, compared to other ecosystems, such as grassland and forest ecosystems, there is a lack of systematic studies on the mechanisms of soil microbial CUE in wetland ecosystems. Several important biogeochemical cycle processes, especially the carbon cycle under anaerobic conditions still need further study. Generally, methane is an important carbon metabolism product in anaerobic environments. Although methane is released at a much slower rate than the rate of peat formation, it is more potent than carbon dioxide in terms of its greenhouse effect [91]. The concentration of CH_4_ in the atmosphere is only one two-hundredth of that of CO_2_, yet its contribution to global warming is about 23%, indicating that the methane metabolism process in anaerobic environments in wetland ecosystems should not be neglected [92]. However, to our knowledge, there are few studies that have explored the effects of flooding on the relationships between methane and soil microbial CUE. For example, Devevre et al. found that flooding has a tendency to decrease the rate of carbon mineralization, whereas it increases methane production [93]. They also confirmed that soil microbial CUE can increase because anaerobic bacteria reuse waste from fermentation during long-term growth. But they have not explored the mechanism of how the methane affects CUE.

## 4. Future Research

Until now, most of the studies have focused on exploring how environmental changes affect CUE in different ecosystems. However, there are still several aspects that need to be focused on in future studies.

### 4.1. Management Patterns

Alternating changes in management patterns in different ecosystems are also important factors affecting soil microbial CUE. Alternating between grazing and the prohibition of grazing significantly affects plant productivity and plant carbon inputs, which might have indirect effects on soil microbial CUE. However, the combined effects of alternation between grazing and the prohibition of grazing on soil microbial CUE are still unclear. For the forest ecosystems, forest gap and thinning are the main management patterns, which could affect the sunlight reaching the soil and the forest soil temperature, thus affecting soil microbial CUE. More attention should be paid to the study of alternating changes in management patterns on soil microbial CUE in future studies.

### 4.2. Strategies of Microorganisms for Adapting to Environmental Change

Microorganisms could adjust their adaptive strategies to reduce the negative effects of environmental changes on their growth, allocating more energy to maintaining their own survival and less energy to growth, which might lead to the reduction of soil microbial CUE. Importantly, climate change has significant impacts on the regional environment, which might also have indirect effects on CUE. Therefore, the mechanism by which the strategies of microorganism adapting to environmental changes affect CUE still need further study, which might be one of the fundamental aspects for better predicting the tendency of terrestrial ecosystems’ carbon cycle under global climate change.

### 4.3. Anaerobic Metabolic Pathways

Methane is the main product of the anaerobic metabolic pathway of the carbon cycle. Especially in wetland ecosystems, the water level can fluctuate frequently, which might even cause the alternation between aerobic and anaerobic conditions. The emergence of anaerobic conditions suggests that the methane should be considered an important component when we calculate the soil microbial CUE. However, the anaerobic metabolic pathway of the carbon cycle is always ignored in the studies of soil microbial CUE in wetland ecosystems. Taken together, there might be at least two more aspects that should be strengthened in future studies: firstly, how to integrate the carbon metabolism pathway of methane into the calculation models of soil microbial CUE in wetland ecosystems and secondly, the mechanisms of how climate change affects methane carbon metabolism pathways.

### 4.4. Microbial Taxonomic Level

Microbial community structure significantly affects soil microbial CUE, which occurs in all ecosystems and is usually characterized by higher CUE in fungi than in bacteria. However, current studies still face limitations in identifying microbial species and their functions. For example, which fungal species play a dominant role in increasing soil microbial CUE and how specific microbial taxa regulate CUE under different environmental conditions still need to be further explored. Future studies should further deepen the understanding of soil microbial CUE at the taxonomic level, especially by identifying specific microbial species and their functional genes to reveal their contributions to CUE. More precise identification of key microbial species involved in carbon transformation, such as certain efficient carbon-sequestration or carbon-decomposition bacteria, can be achieved through advanced techniques. By combining taxonomic ecology, we expect to reveal the complex relationship between microbial diversity and CUE.

## 5. Conclusions

We found that the effects of environmental factors on soil microbial CUE in different ecosystems are complex. Future research should prioritize the following aspects: the influence of management patterns on CUE, effects of changes in the strategy of microorganisms adapted to the environment on CUE, effects of anaerobic metabolic pathways, especially in wetland ecosystems, and effects of microbial taxonomic level.

## Figures and Tables

**Table 1:** Comparison of the calculation methods for the soil microbial carbon use efficiency.

Method	Stoichiometric Modeling	^13^C Glucose Tracing	^18^O Water Tracing
Principle	Based on stoichiometric ratios	Based on biomass variation	Based on growth rate variation
Formula	$\mathrm{CUE}={\mathrm{CUE}}_{\max}\times {\left[\frac{\left(S_{C:N}\;\times {\;S}_{C:P}\right)}{\left(K_{C:N}+S_{C:N}\right)\;\times \;\left(K_{C:P}+S_{C:P}\right)}\right]}^{0.5}$	CUE=MBC13MBC13+R13	$\mathrm{CUE}=\frac{C_{\mathrm{growth}}}{\left(C_{\mathrm{growth}}+\mathrm{Respiration}\right)}$
Substrate	——	^13^C-Glucose	^18^O-H_2_O
Labeled	No	Yes	Yes
Incubation time	——	Short-term	Short-term
Advantages	No cultivation is required and it can be measured directly	Simple and easy to use	Measures the rate of microbial growth directly
Disadvantages	Model assumptions, obtained from empirical coefficients	Microbial biomass needs to be measured and is sensitive to changes over time	Only suitable for short-term experiments, stable over time
Carbon use efficiency (CUE)	The maximum value of the CUE is 0.6, with a small fluctuation range [31]	(1) CUE may be underestimated [28,32] (2) CUE may be overestimated [33,34]	CUE may be underestimated [35,36]

Notes: Carbon use efficiency (CUE), ^13^C glucose tracing (^13^C), ^18^O water tracing (^18^O), microbial biomass (MBC), and respiration (R). K_C:N_ and K_C:P_ are the half–saturation constants for CUE based on the stoichiometry of the substrate C:N and C:P effectiveness, and B_C:X_ is the elemental C:N or C:P ratio of the microbial biomass, where S_C:N_ and S_C:P_ represent the scalars of resource availability for microbial growth based on the stoichiometries of extracellular enzyme activities and soil.

## Data Availability

Not applicable.

## Abbreviations

The following abbreviations are used in this manuscript:

CUE	microbial carbon use efficiency
BG	β-1,4-glucosidase
LAP	leucine aminopeptidase
NAG	β-1,4-N-acetylaminoglucosidase
AP	acid phosphatase
MBC	microbial biomass carbon
R	respiration
C	carbon
N	nitrogen
P	phosphorus
CO_2_	carbon dioxide
CH_4_	methane
